# Utility of paired plasma and drainage fluid mNGS in diagnosing acute intra-abdominal infections with sepsis

**DOI:** 10.1186/s12879-024-09320-1

**Published:** 2024-04-17

**Authors:** Jia-yu Mao, Dong-kai Li, Dong Zhang, Qi-wen Yang, Yun Long, Na Cui

**Affiliations:** 1 Department of Critical Care Medicine, State Key Laboratory of Complex Severe and Rare Diseases, Peking Union Medical College Hospital, Chinese Academy of Medical Science and Peking Union Medical College, 100730 Beijing, China; 2 Department of Clinical Laboratory, Peking Union Medical College Hospital, Chinese Academy of Medical Science and Peking Union Medical College, Beijing, China

**Keywords:** Sepsis, Acute intra-abdominal infection, mNGS, Plasma and peritoneal drainage, Antibiotics

## Abstract

**Background:**

Metagenomic next-generation sequencing (mNGS) has been increasingly applied in sepsis. We aimed to evaluate the diagnostic and therapeutic utility of mNGS of paired plasma and peritoneal drainage (PD) fluid samples in comparison to culture-based microbiological tests (CMTs) among critically ill patients with suspected acute intra-abdominal infections (IAIs).

**Methods:**

We conducted a prospective study from October 2021 to December 2022 enrolling septic patients with suspected IAIs (*n* = 111). Pairwise CMTs and mNGS of plasma and PD fluid were sent for pathogen detection. The mNGS group underwent therapeutic regimen adjustment based on mNGS results for better treatment. The microbial community structure, clinical features, antibiotic use and prognoses of the patients were analyzed.

**Results:**

Higher positivity rates were observed with mNGS versus CMTs for both PD fluid (90.0% vs. 48.3%, *p* < 0.005) and plasma (76.7% vs. 1.6%, *p* < 0.005). 90% of enrolled patients had clues of suspected pathogens combining mNGS and CMT methods. Gram-negative pathogens consist of most intra-abdominal pathogens, including a great variety of anaerobes represented by *Bacteroides* and *Clostridium*. Patients with matched plasma- and PD-mNGS results had higher mortality and sepsis severity. Reduced usage of carbapenem (30.0% vs. 49.4%, *p* < 0.05) and duration of anti-MRSA treatment (5.1 ± 3.3 vs. 7.0 ± 8.4 days, *p* < 0.05) was shown in the mNGS group in our study.

**Conclusions:**

Pairwise plasma and PD fluid mNGS improves microbiological diagnosis compared to CMTs for acute IAI. Combining plasma and PD mNGS could predict poor prognosis. mNGS may enable optimize empirical antibiotic use.

## Background

Intra-abdominal infections (IAI) are a major source of sepsis and mortality in critical ill patients [1]. Rapid and accurate diagnosis of infecting pathogens is essential to optimize clinical management and improve outcome of septic patients [2]. Intra-abdominal culture and antibiotic susceptibility test may help to ensure effective anti-infective therapy and minimize excessive use of broad-spectrum antibiotics [3]. However, the sensitivity of peritoneal drainage (PD) culture varies between 19.5 and 63.7% in clinical settings [4, 5], moreover conventional culture methods require a certain period of time for examination [6]. Therefore, alternative diagnostic strategies, such as metagenomic next-generation sequencing, could complement conventional culture-based methods to enhance infection control measures [7]. 

Metagenomic next-generation sequencing (mNGS) is a highly efficient tool for pathogens detection through sequencing of random nucleic acids in samples [8, 9]. Since the performance of PD fluid mNGS has been verified in a certain number of reports [10, 11], plasma mNGS alone is not sufficient for a positive detection rate, and pairwise plasma and PD mNGS may improve pathogen detection and the underlying pathogenic mechanism. Our study aimed to comprehensively analyze pairwise plasma and PD mNGS for diagnosis and treatment compared with CMTs in critical care patients.

## Methods

### Patients and study design

This single-center prospective observational study was conducted in the ICU (Intensive Care Unit) department of the Peking Union Medical College Hospital (PUMCH), which is a tertiary academic hospital. Septic patients with severe acute IAI requiring ICU care from the emergency department were admitted from October 2021 to December 2022. Acute IAI were diagnosed according to previous guidelines [12–14], patients with source control procedures like surgery or percutaneous puncture drainage undergone on the first day of admission were enrolled in this study. The exclusion criteria were as following: (1)<18-year-old; (2) ICU stay ≤ 24 h; (3) incomplete clinical data, like missing data such as admission diagnosis, antimicrobial therapy records, without access to drainage fluid, etc.; (4) refusal to undergo mNGS detection; and (5) failure to obtain written consent. The ethics committee of PUMCH approved this study (approval number: JS-1170). Informed consent was obtained from the next kin of all patients, and the study was registered with the Chinese Clinical Trial Registry (identifier ChiCTR-ROC-17,010,750).

In addition, to investigate the influence of mNGS on clinical treatment, a retrospective cohort (non-mNGS group) was included for comparison with the prospective mNGS group. The non-mNGS group comprised patients with acute IAIs admitted to our department from August 2020 to October 2021, before the standardized implementation of mNGS testing for peritoneal drainage fluid samples at our center. All patients in both groups met the same inclusion and exclusion criteria and received empiric antibiotic therapy according to relevant guidelines. The comparison between the two groups aimed to assess the impact of mNGS results on antibiotic utilization patterns.

Initial empirical medications in both these two groups were administered according to relevant IAI guidelines [15, 16]. Empiric antibiotic therapy should include agents against aerobic Gram-negative bacteria (e.g., Enterobacteriaceae), aerobic streptococci, and obligate enteric anaerobic organisms. Resistant or opportunistic pathogens such as *Candida spp.* should also be covered in selected conditions like a healthcare setting, corticosteroid use, organ transplantation or previous antimicrobial therapy. Antibiotic therapy was also selected based on based on infection`s severity, community or hospital acquisition, presence of septic shock, and the degree of peritoneal observed during surgery [12, 17, 18]. Empiric antimicrobial therapy should be narrowed once culture and susceptibility results are available and adequate clinical improvement is noted. In mNGS group, the therapeutic regimens were adjusted based on mNGS detection, such as lack of evidence of certain bacteria may indicate early discontinuation of corresponding treatment. The retrospective non-mNGS group collected septic patients with acute IAI admitted to our department from August 2020 to October 2021. The inclusion and exclusion criteria were consistent with the above. Only CMTs of plasma and PD was sent for pathogen detection. All enrolled patients underwent the same diagnosis and treatment procedure in our study.

### Data collection

Clinical history evaluation and laboratory tests were carried out, including age, sex, routine blood examination, procalcitonin (PCT), hemodynamic parameters, respiratory parameters, Sequential Organ Failure Assessment (SOFA) score, Acute Physiology and Chronic Health Evaluation (APACHE) II score and experiential antibiotic use upon ICU admission. Duration of ICU stay time and 28-day mortality rate were recorded as follow-up data. Peripheral blood samples and intraperitoneal fluid samples were obtained for mNGS analysis and culture-based microbiological tests immediately after source control procedures. At least 5mL of blood sample and PD fluid was collected for adequate microbiological testing, and samples were sent to laboratory immediately avoiding influence on results.

According to procedures of the central laboratory of the Clinical Laboratory Department PUMCH, blood and PD fluid culture and confirmation of species identification were performed. For blood culture, standard aerobic and anaerobic bottles were processed by the microbiology laboratory according to standard protocol using Bac T Alert,5 a continuously monitored, carbon dioxide detection system. Specimens were incubated for 7 days and all positive vials were inoculated onto appropriate media and further processed by culture-based techniques [19]. For PD fluid culture, the sample was performed using routine isolation media, including blood agar, eosin methylene-blue (EMB) agar, Mueller-Hinton agar and cooked meat medium (Oxoid, UK). Plates were incubated at 37 °C in 5–10% CO2 for 24–48 h using BC60 automated culture system (Autobio Diagnostics Co., Ltd., China). The strains were then isolated and identified the species using the VITEK-2 Compact Instrument (bioMérieux, France).

Plasma and PD fluid of each patient were obtained to perform metagenomic next-generation sequencing and data analysis. The mNGS detection process includes host cell removal, nucleic acid extraction, library preparation, sequencing, and bioinformatics analysis. Briefly, DNA was extracted using a DNA extraction kit (Tiangen Biotech (Beijing) Co., Ltd., China) in a 1 mL sample. cDNA was generated using reverse transcriptase and dNTPs (Thermo Fisher). Libraries were constructed for the DNA and cDNA samples using a Nextera XT DNA Library Prep Kit (Illumina, San Diego, CA). Library pools were loaded onto the Illumina Nextseq CN500 sequencer for 75 cycles of single-end sequencing, generating approximately 20 to 40 million reads for each library. Quality control and evaluation of FASTQ format data obtained by sequencing were carried out, and low-quality or undetected sequences, sequences contaminated by splices, high-coverage repeats, and short read-length sequences were filtered to retain high-quality sequencing data. Microorganism identification was obtained through mapping to the commercial pathogen database as previously described [20]. In the mNGS analysis, the relative abundances of microbial taxa were determined and compared to predefined abundance cut-offs based on previous studies to differentiate likely pathogens from commensals/contaminants. Low-abundance organisms were excluded from analysis. The sequencing data are available at the NCBI SRA database via BioProject accession PRJNA987137.

### Outcomes assessment

The primary outcome was the difference in microbiologic positivity rates between mNGS and conventional cultures for PD fluid and plasma. Secondary outcomes included the concordance between plasma and PD mNGS results and their correlation with clinical parameters, the microbiota profiles in intra-abdominal infections, and the impact of mNGS on empirical antimicrobial therapy in terms of agent selection and duration.

### Statistical analysis

Continuous variables were presented as the mean ± standard deviation or median with a range and analyzed by Student’s t test or the rank-sum (Mann–Whitney U) test. Categorical variables were presented as proportions (absolute and relative frequencies) and analyzed by the chi-square test or Fisher’s exact test. Statistical analysis was conducted by the statistical software SPSS version 24 (IBM Corp., Armonk, NY, United States). Differences with values of *p* < 0.05 were defined statistically significant.

## Results

### Basic characteristics

**Fig. 1:**
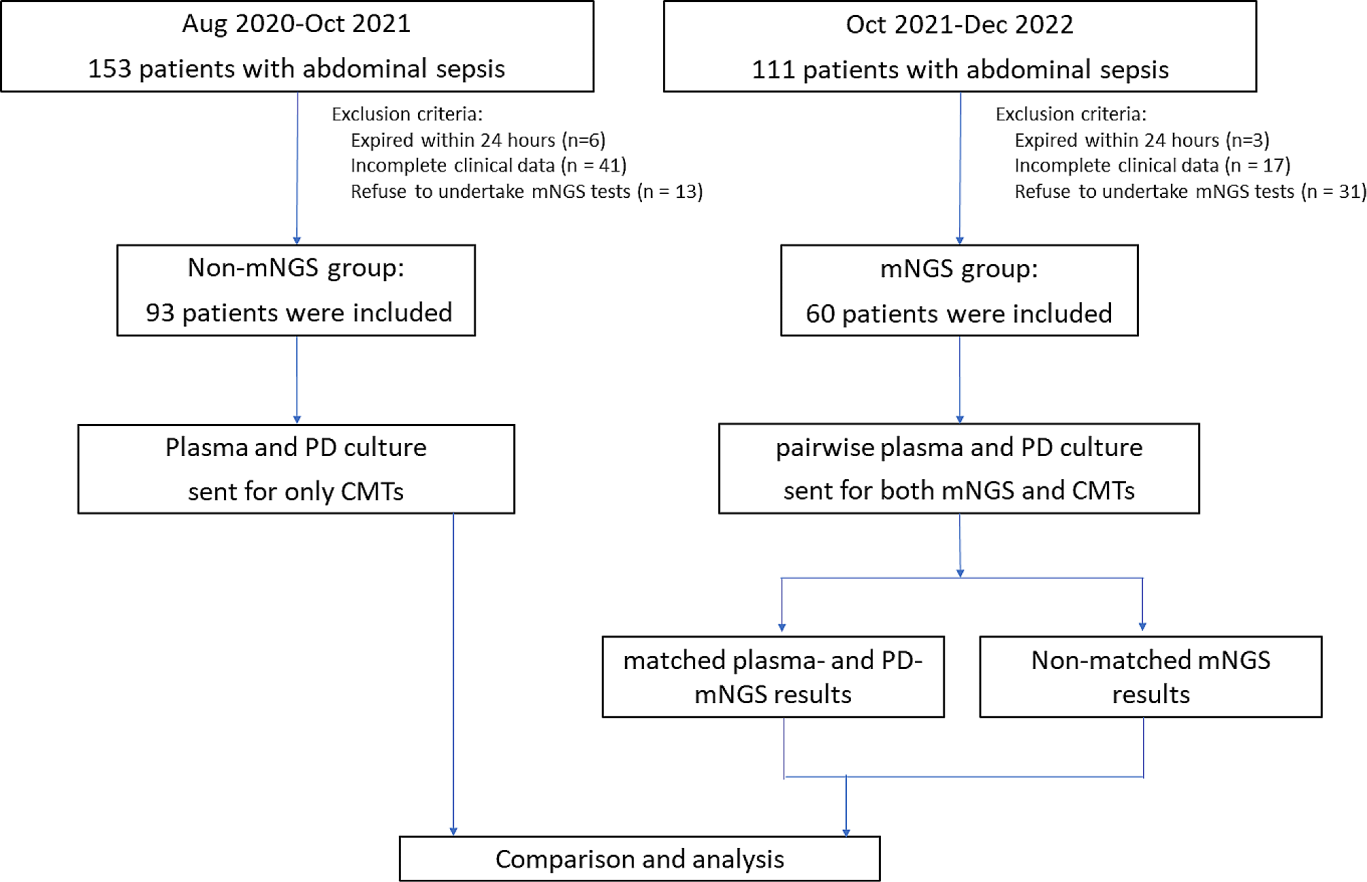
Flowchart showing step-by-step selection of patients included in the study

From October 2021 to December 2022, a total of 111 septic patients were diagnosed with acute IAI and included in our study. Among them, 17 patients without access to drainage fluid were excluded, 31 were excluded since they refused to undergo mNGS tests, and 3 patients that did not survive at least 24 h were also excluded, as shown in Fig. 1. Therefore, 60 patients were included in the research. The basic characteristics of all the patients enrolled were shown in Table 1. The most common etiology was intestinal perforation or obstruction.

**Table 1:** The General Characteristics of the Patients Included in This Study

Characteristics	*n* = 60
Age, yr	62.1 ± 14.1
Gender, n (%)	
Male	31(51.7)
Basic disease, n (%)	
Hypertension	18(30)
Diabetes mellitus	17(28.3)
Chronic kidney disease	4(6.7)
Coronary infarction	5(8.3)
Rheumatic disease	2(3.3)
State of nutrition	
Body Mass Index, kg/m2	23.4(20.2–26.7)
Albumin, g/L	28.3(22.4–34.1)
Etiology, n (%)	
Intestinal perforation or obstruction	55(91.7)
Biliary tract disease	2(3.3)
Skin and soft tissue	2(3.3)
Others	1(1.7)
Presence of septic shock	34(56.7)
Type of source control	
Surgical	49(81.7)
Radiologic	11(18.3)

### Microbiological analytic performance of PD culture and mNGS

**Fig. 2:**
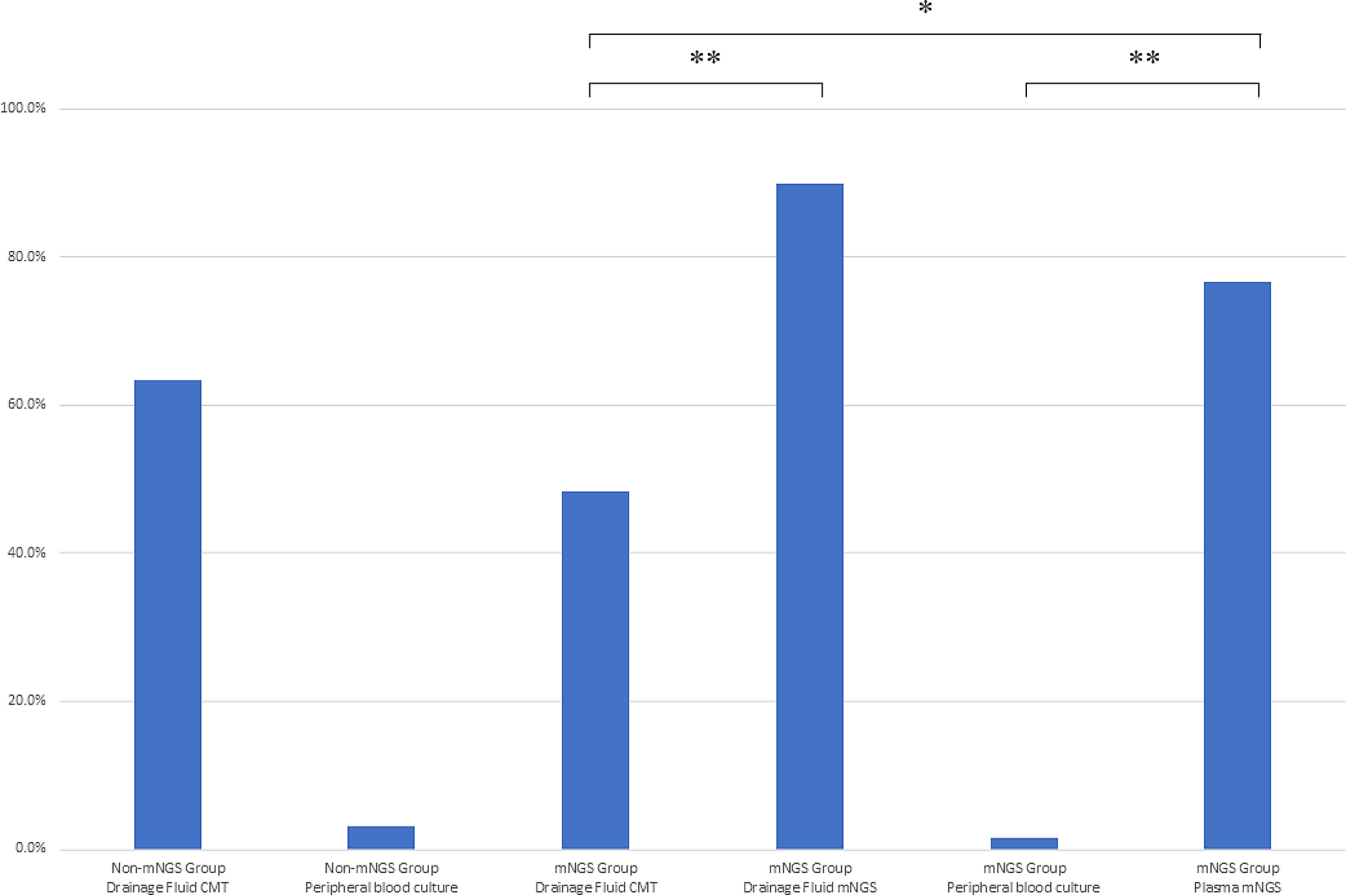
Comparison of positive rate of different diagnostic methods in the study

**Fig. 3:**
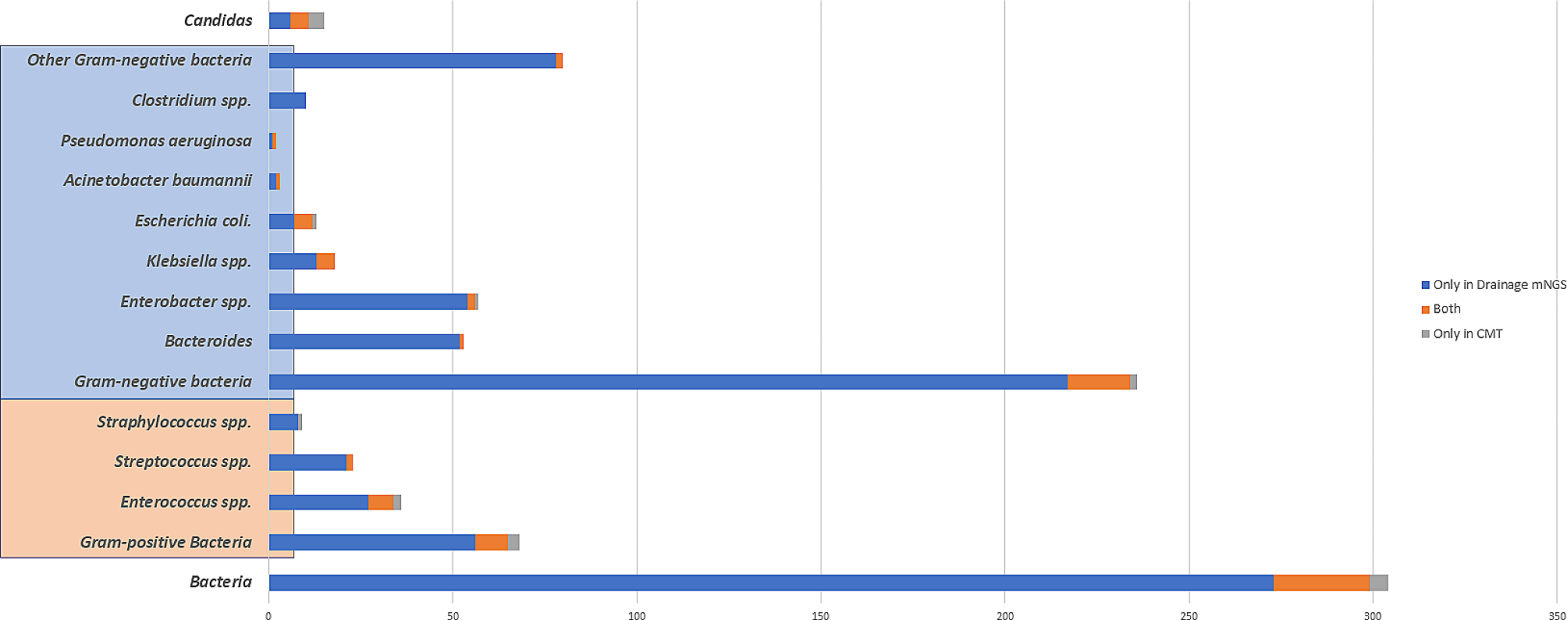
Distribution of positive and matched results of drainage fluid mNGS and CMT according to the detected pathogenic strains

Samples of PD fluid was sent for both mNGS and CMTs, then their microbiological diagnostic performance was compared. The positivity rate of PD mNGS was significantly higher than that of CMT culture (90.0% vs. 48.3%, *p* < 0.001), as shown in Fig. 2. Overall, 90% of patients (54/60) had suspected pathogens detected by combining mNGS and the CMT method of drainage fluid. mNGS detected a higher number of bacterial pathogens (299 strains) compared to CMTs (31 strains) in PD fluid samples, with the majority of the pathogens detected exclusively by mNGS being gram-negative bacteria (217 strains). We found that *Enterobacter spp.* (56[93.3%]) was the most commonly isolated bacteria by PD mNGS in our study, followed by *Bacteroides* (53[88.3%]) and *Enterococcus spp.* (34[56.7%]). In addition, *Clostridium spp.* was detected only through mNGS compared with culture-based diagnostics. Furthermore, positive results for fungus (11 strains in mNGS vs. 9 strains in PD culture) were consistent in different groups, as shown in Fig. 3.

In the pathogenic detection of PD fluid, 35 patients (58.3%) had at least one matched mNGS and CMT result, and among them, 9 patients (15%) had a complete matched result. Approximately half of the mNGS results matched the corresponding CMT results based on the pathogenic strains (gram-negative: 17/19; gram-positive: 9/12; Fungi: 5/9).

### Microbiological analytic performance of plasma mNGS and its comparison with PD mNGS

**Fig. 4:**
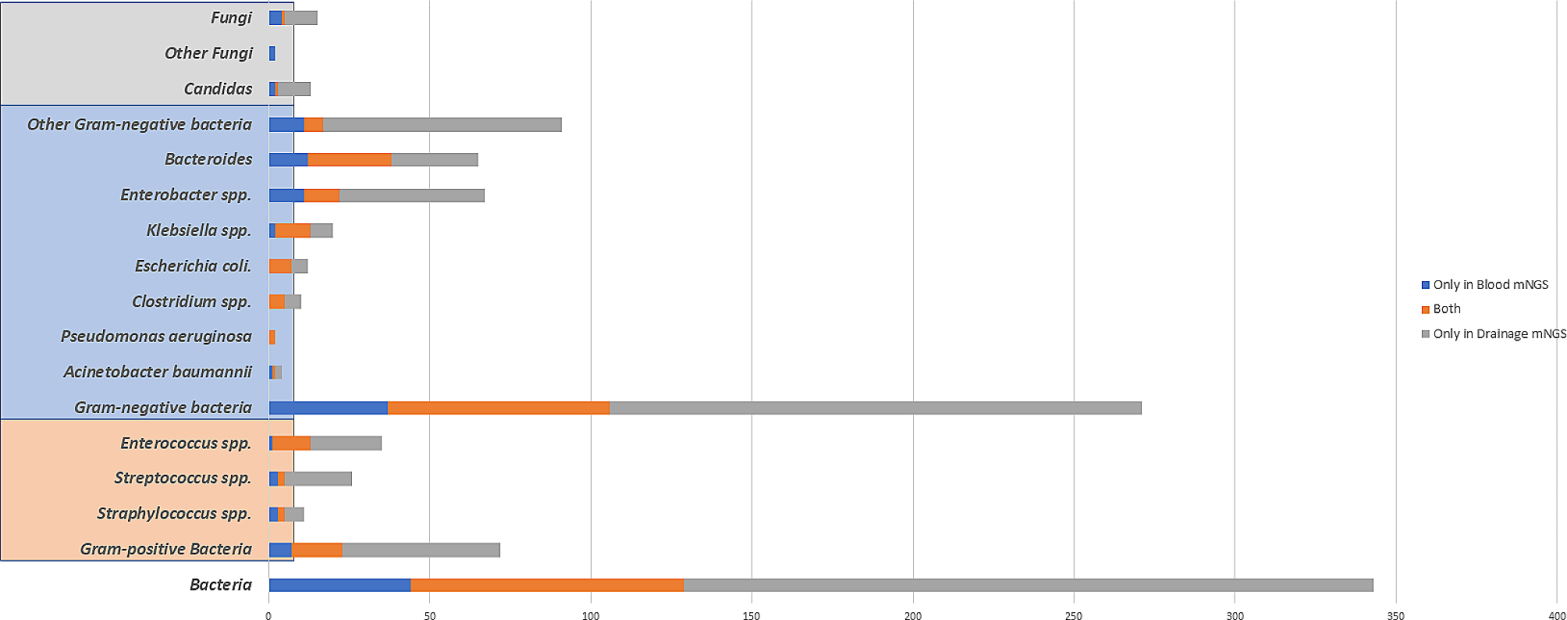
Distribution of positive and matched results of mNGS on plasma and drainage sample according to the detected pathogenic strains

The positivity rate of plasma mNGS (46/60, 76.7%) was significantly higher than that of peripheral blood culture (1/60, 1.6%, *p* < 0.005) and even higher than that of PD culture (29/60, 48.3%, *p* < 0.01). Similar to the findings in PD fluid, plasma mNGS primarily contributed to the detention of gram-negative bacteria from plasma mNGS. The most commonly isolated bacteria by plasma mNGS were *Bacteroides* (12 [20%]) and *Enterobacter spp.* (11 [18.3%]). Based on the pathogenic strains, a portion of the plasma mNGS results matched the corresponding PD mNGS results (gram-positive: 16/65; gram-negative: 69/234; fungi: 1/11). We found that *Klebsiella spp.* (11/18), *Escherichia coli.* (7/12), *Clostridium spp.* (5/10) and *Bacteroides* (26/53) were more likely to be detected from both plasma and PD mNGS, as shown in Fig. 4.

### Clinical interpretation of mNGS

Patients were divided into different subgroups through positive plasma and PD mNGS with corresponding matched results. Twenty-nine patients (48.3%) had at least one matched plasma and PD mNGS result. In the mNGS-matched group, more patients exhibited septic shock (61% vs. 47.4%, *p* = 0.0169). Overall, mortality, duration of ICU stays, SOFA scores, vasoactive usage duration and mechanical ventilation duration were significantly higher in the matched group. The matched group also showed significantly higher WBC (white blood cells) level, as shown in Table 2. We also compared the reporting time of mNGS and CMT. The results showed that, compared with the mNGS reporting time (34.9 ± 7.0 h), the levels of the PD culture reporting time (53.7 ± 13.7 h) showed statistically significant differences (*p* < 0.0001).

**Table 2:** Characteristics of patients for subgroup analysis

Characteristics	Matched mNGS group *n* = 29	Non-matched mNGS group *n* = 31	*p*
Age, yr	66.1 ± 16.5	59.8 ± 11	0.0918
Gender, n (%)			
Male	19(65.5)	15(48.4)	0.1869
APACHEII	20.6 ± 7.2	19.2 ± 7.0	0.4709
SOFA	8.1 ± 2.7	6.3 ± 2.7	0.0130^*^
Mortality, n(%)	5(17.2)	0(0)	0.0153^*^
ICU stay time, d	19(11-30.4)	8.5(6.3–19.0)	0.0205^*^
Septic shock	21(61.0)	13(47.4)	0.0169^*^
Vasoactive usage duration, h	37.2(0-102.6)	0(0–23.0)	0.0134^*^
MV duration, h	35.8(3.6-117.5)	5.4(0-20.7)	0.0077^**^
WBC, *10^9^/L	13.5 ± 8.7	9.6 ± 5.2	0.049^*^
PCT, ng/mL	1.2(0.44–5.2)	2.9(0.81–8.4)	0.2075

mNGS, metagenomic next-generation sequencing; APACHE II, Acute Physiology and Chronic Health Evaluation II score; SOFA, Sequential Organ Failure Assessment score; ICU, Intensive Care Unit; MV, mechanical ventilation; WBC, white blood cell; PCT, procalcitonin

**Fig. 5:**
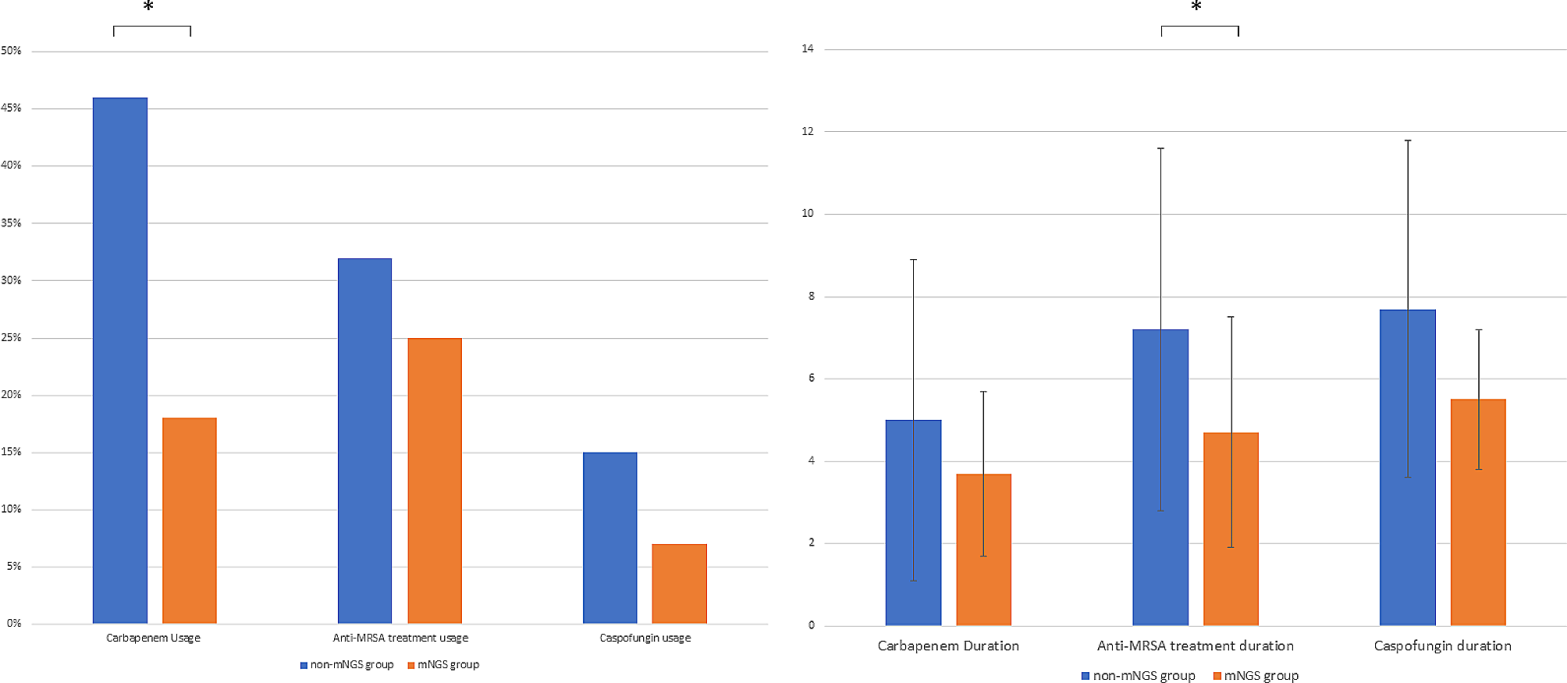
Comparison between the non-mNGS and mNGS groups on the empirical carbapenem, anti-MRSA and caspofungin usage. Left: Patients received the corresponding treatment. Right: Duration of the corresponding treatment. Y-axis, hour

Empirical medications such as carbapenem, vancomycin, caspofungin etc. were administered after all patients admitted. In our prospective study, the therapeutic regimens were adjusted based on mNGS detection for better treatment. Usage of antibiotics and its duration compared with the retrospective study (non-mNGS group) was undertaken. The usage of carbapenem was significantly reduced in the mNGS group (30.0% vs. 49.4%, *p* < 0.05), as shown in Fig. 5. Moreover, a significantly shorter duration of anti-MRSA treatment (5.1 ± 3.3 vs. 7.0 ± 8.4, *p* < 0.05) was shown in the mNGS group showed compared with the non-mNGS group.

## Discussion

In this study, the microbiological diagnostic performance of mNGS for acute IAI with sepsis and its clinical impact on treatment were assessed. The microbiological community structure, clinical features, and prognoses of the patients enrolled were analyzed. Pairwise mNGS tests on plasma and drainage fluid were combined to further interpret acute IAI with sepsis.

IAI is the second most common site of sepsis which is a leading cause of mortality in ICU [21]. Microbiological analyses are of great importance for initial antibiotic strategy [22]. However, the culture-based microbiological test requires lengthy experimental time that may delay selection of appropriate antibiotic [1]. With the advancement of sequencing technologies, culture-independent technologies such as the mNGS test have shown superior diagnostic performance in infectious disease [23]. Diagnostic sensitivity was significantly increased by mNGS compared to conventional cultures, even after antibiotic treatment [9, 24]. In recent years, mNGS has been widely applied in diagnosing sepsis [23], bloodstream infections [25], pneumonia [9] and pediatric infections [26]. PD mNGS has also been verified in a certain number of reports [10, 11]. Moreover, pairwise plasma and PD mNGS may improve pathogen detection and the underlying pathogenic mechanism.

In our study, higher positive rates of pathogen detection of mNGS were shown compared to culture-based microbiological test in both PD and plasma samples. Consistent with previous reports, the percentage of positive PD cultures in our study were 48.3%, and PD mNGS could significantly improve the positive diagnostic rate of PD samples. Moreover, 90% of enrolled patients discovered clues of suspected pathogens through PD combined with mNGS and CMTs. Based on the detected pathogenic strains, gram-negative bacteria had the tendency to be detected through mNGS, especially a great variety of anaerobes represented by *Bacteroides* and *Clostridium*. These results suggest that we should pay more attention to antibiotic selection for anaerobic bacteria. The mNGS assay and culture-based microbiological test of PD showed high consistency in pathogen detection.

In addition, our previous study showed that the plasma mNGS was more rapid compared with PD culture, early microbiological diagnosis resulted timely and accurate initial antibiotic treatment for IAI with sepsis [20]. The results of plasma mNGS and its comparison with PD microbiological analyses showed us more interpretation of IAI in this research. Almost half of the positive plasma mNGS results and the corresponding PD mNGS were consistent. We may guess that severe IAI may accompany with pathogens spread into the blood [10]. Plasma mNGS can assist in convenient pathogen detection besides PD fluid [27, 28]. Nevertheless, mNGS exerted superior sensitivity to traditional peripheral blood culture. Pathogens detected in both plasma and PD mNGS may be more instructive in clinical practice. Moreover, in our study, patients with matched plasma and PD mNGS results seemed to exhibit a higher incidence of mortality, septic shock, and organ failure. Therefore, matched of plasma and PD mNGS results may act as an early warning indicator.

Due to the complexity of IAI, polymicrobial infections and increasing presence of multidrug-resistant (MDR) pathogens both need clinical attention. Adequate coverage of antibiotic and timely de-escalation are both important [29]. In our study, the therapeutic regimens were adjusted based on mNGS detection for better treatment. The use of antibiotics and their duration were compared between the two cohorts. Benefitting from the rapid feedback of mNGS, less usage of carbapenem in the mNGS group was discovered. Nevertheless, with more sensitive information on pathogen detection, a significantly shorter duration of vancomycin, which is anti-MRSA (methicillin-resistant Staphylococcus aureus) treatment was also shown in the mNGS group. Whether to initiate therapy against MRSA was emphasized in the SSC guidelines on management of sepsis and septic shock of 2021 [17]. Since both failure covering MRSA in a patient with MRSA and unnecessary MRSA coverage in a patient without MRSA may be harmful. Overall, pairwise plasma and PD mNGS detection in our study seemed to be associated with less use and shorter duration of broad-spectrum antibiotics, which may help reduce antibiotic resistance and improve prognosis.

Our study still had some limitations. First, our study was a relatively small single-center study, and a larger multicenter cohort should be conducted to provide more information for clinical practice. What`s more, subgroup analysis is significant based on infection sites and microbiological spectrum. Second, a randomized controlled intervention study is still needed to verify the causal relationship between the use of mNGS and the reduced use of broad-spectrum antibiotics. Furthermore, the clinical implications and interpretation of drug resistance gene testing results obtained through mNGS warrant further investigation.

## Conclusions

In summary, our study demonstrated that pairwise plasma and drainage mNGS can improve the positive diagnostic rate of suspected pathogens in acute IAI with sepsis. A combination of plasma and PD mNGS may indicate early warning of poor prognosis and change clinical practice. The application of mNGS has the potential to optimize the empirical usage of antibiotics.

## Data Availability

The sequencing data are available at the NCBI SRA database via BioProject accession PRJNA987137.

## Abbreviations

mNGS: Metagenomic next-generation sequencing
PD: peritoneal drainage
CMTs: culture-based microbiological tests
IAI: intra-abdominal infection
ICU: Intensive Care Unit
PUMCH: Peking Union Medical College Hospital
PCT: procalcitonin
APACHE II: Acute Physiology and Chronic Health Evaluation II
SOFA: Sequential Organ Failure Assessment
WBC: white blood cells
MRSA: methicillin-resistant Staphylococcus aureus
MDR: multidrug-resistant
